# Rapid Detection of *Staphylococcus aureus* from Gym Environments for Health Risk Monitoring Using Printed Nanochains-Based Biosensors

**DOI:** 10.3390/bios15120791

**Published:** 2025-12-01

**Authors:** Liang Huang, Shidong Hu, Zhicheng Zheng, Yaxin Li, Maolin Xu, Zeying Zhang, Jingqun Cheng, Yujing Zhang, Yonggan Xue, Meng Su, Xiaohui Du

**Affiliations:** 1 Senior Department of General Surgery, The First Medical Center of Chinese PLA General Hospital, Beijing 100853, China; hlsurgery@126.com (L.H.); hushidong2014@163.com (S.H.); yongganxue@163.com (Y.X.); 2 Department of General Surgery, Beijing Tiantan Hospital, Capital Medical University, Beijing 100070, China; 05zhengzc2025@mail.ccmu.edu.cn; 3 College of Chemistry and Molecular Sciences, Henan University, Kaifeng 475004, China; l13373769327@163.com; 4 Department of Emergency Medicine, The First Medical Center of Chinese PLA General Hospital, Beijing 100853, China; dushubaoguo@163.com; 5 Key Laboratory of Green Printing, Institute of Chemistry, Chinese Academy of Sciences/Beijing Engineering Research Center of Nanomaterials for Green Printing Technology, Beijing National Laboratory for Molecular Sciences (BNLMS), Beijing 100190, China; zhangzy@iccas.ac.cn (Z.Z.); chengjq@iccas.ac.cn (J.C.); 6 School of Management, Beijing Sport University, Beijing 100084, China; zyujing1@126.com

**Keywords:** *Staphylococcus aureus*, polystyrene nanoparticles, optical biosensor, real sample, specific detection

## Abstract

Gyms are indoor environments in which many people perform physical exercise and could potentially increase the risks of bacterial contamination and dissemination. *Staphylococcus aureus* (*S. aureus*) is one of the most prevalent bacteria in community-acquired infections; thus, the rapid detection and continuous monitoring of *S. aureus* are crucial for evaluating the hygienic status of gym environments. This work describes the fabrication of a nanochain-based biosensor for *S. aureus* detection using carboxyl-modified polystyrene (PS) nanoparticles functionalized with a specific antibody. When target bacteria bind to the nanochains, they yield distinct color changes which support the directly visualizable analysis of optical images, recorded using optical microscopy or even a smart mobile phone. In addition to high portability, this biosensor is also capable of the quantification and continuous monitoring of the bacterial load in a gym environment over a broad linear range (10^0^ CFU/mL~10^5^ CFU/mL), with a detection limit of 1 CFU/mL. In summary, this study validated the applicability of the biosensors for the rapid detection and real-time monitoring of gym environmental pathogens.

## 1. Introduction

Large numbers of gyms have been established in communities, workplaces, and universities worldwide to provide convenience for people’s exercises; thus, the hygienic status of gym environments directly impacts users’ health and safety. *S. aureus*, one of the most prevalent bacteria in both communities and healthcare facilities [1,2], colonizes on the skin, nares, and mucous membranes of healthy individuals [3,4]. The characteristics of indoor gyms, like high-density human traffic, frequent equipment use, and humid thermal environment, significantly potentiate *S. aureus* spread and colonization [5,6,7]. *S. aureus* poses multiple threats to human health which can cause a wide range of infections, from localized to systemic. Localized infections include skin and soft tissue infections, such as folliculitis and wound infections [8]. More severely, it may invade deep tissues or the bloodstream, triggering life-threatening conditions like pneumonia [9], bacteremia [10], and endocarditis [11]. The emergence of Methicillin-resistant *S. aureus* (MRSA) has made treatment extremely challenging. Due to its resistance to multiple commonly used antibiotics, MRSA often leads to prolonged illness, increased treatment costs, and higher mortality rates [12,13]. In summary, *S. aureus* is not only a frequent source of infection but also a significant public health challenge. Therefore, the systematic monitoring of the *S. aureus* contamination level in public indoor environments like gyms is critically important for public health, outbreak prevention, and curbing resistant pathogen spread.

Currently, selective culture-based methods remain the gold standard for pathogen detection [14]; however, they are time-consuming (requiring 2–5 days) and exhibit a limited sensitivity because they lack the capability to detect viable but non-culturable (VBNC) bacteria [15]. Molecular detection techniques, such as quantitative polymerase chain reaction (qPCR) techniques [16] and Loop-mediated isothermal amplification (LAMP) [17], show a high sensitivity and reduce the assay time to hours, while they require complex nucleic acid extraction procedures which are generally labor-intensive. The other most common methods, like the enzyme-linked immunosorbent assay (ELISA) [18], also face the burden of high cost, time consumption, and complex procedures. Single-cell Raman spectroscopy [19,20] enables culture-free, rapid (<1 h) pathogen detection by characterizing bacteria phenotypic characteristics. However, it relies on standardized spectral databases, and its detection accuracy can be compromised by sample matrices. 16S ribosomal RNA gene (16S rRNA) sequencing [21,22] is considered as the gold standard method for microbial species-level identification. However, due to the need for external laboratory testing, it inevitably prolongs the report time of results (>48 h) and increases the cost. In recent years, several microfluidic chips [23,24] and biosensors [25,26] were developed to meet the demands for speed, accuracy, and high-throughput bacteria detection. For instance, a finger-actuated CRISPR-Cas12a microfluidic chip [27] was developed for the multiplex detection of seven pathogens, with a limit of detection 500 CFU/mL, and reported in 62 min. Xie et al. [28] proposed a digital microfluidic platform for integrated pathogen detection, delivering results within 55 min. In recent years, various nanoparticles have been employed as desirable materials to fabricate biosensors for bacteria detection. For example, metal nanoparticles (silver [29] and gold [30]) induce color changes through surface plasmon resonance (SPR), and magnetic nanoparticles [31] enable the concentration of target bacteria via magnetic separation. Nevertheless, the sample processing in these studies needs protein extraction or nucleic acid extraction, which demands professional expertise. Moreover, they often require the precise adjustment of detection signals using complex instruments which limit their widespread application.

In this work, we developed an optical biosensor that can directly detect and monitor pathogenic bacteria in gym environments. Carboxyl-modified polystyrene (PS) nanoparticles were selected to assemble nanochains because they are full of carboxyl groups, which are easily functionalized with specific antibodies. After antibody incubation, a bovine serum albumin (BSA) solution was used to block non-specific carboxyl sites to ensure specificity. Only 50 µL of the sample was needed in this detection experiment. When samples were added on this biosensor, the effective preconcentration of candidate bacteria toward the biosensors surface worked to improve sensitivity (limit of detection: 10^0^ CFU/mL) and shorten the reaction time to 10 min. Once *S. aureus* was captured by the nanochains, it triggered distinct color changes that can be observed with the unaided eye through an optical microscope, and even a smartphone. This work demonstrated a simple, rapid, and culture-free diagnosis tool for the visualizable detection and monitoring of *S. aureus* in gym environments.

## 2. Experimental Section

### 2.1. Materials and Apparatus

Carboxyl-modified polystyrene nanoparticles (diameter: 500 nm) were purchased from the Huge Biotechnology Co., Ltd. (Shanghai, China). Sodium dodecyl sulfate (SDS) was obtained from J&K Scientific (Marbach, Germany). The N-hydroxysuccinimide (NHS), N-ethyl-N′-(dimethylaminopropyl) carbodiimide (EDC), and bovine serum albumin (BSA) were purchased from Thermo Fisher Scientific (Waltham, MA, USA). Phosphate-buffered solution (PBS, PH = 7.2–7.4) was purchased from Beijing Solarbio Science & Technology Co., Ltd. (Beijing, China). The *E. coli*, *S. epidermidis*, and *S. aureus* standard bacteria were obtained from BeNa Culture Collection (Xinyang, China). The anti-*S. aureus* antibody was purchased from the Beijing Bioss Biotechnology Co., Ltd. (Beijing, China), with a concentration of 1 mg/mL.

The optical images were captured using an optical microscope (Nikon, LV100, Tokyo, Japan) or smartphone (Oppo FindX3 Pro, Oppo, Dongguan, China). The SEM images were recorded using a scanning electron microscope (HITACHI, S-4800, Tokyo, Japan). The white light source was produced by a xenon lamp (Beijing China Education Au-Light Technology Co., Ltd. CEL S500, Beijing, China), with an adjustable polarizer (Nikon, LV-PO). The scattering response of the nanochains was analyzed using a monochromator and a CCD detector (iDus DU420A-OE, Oxford Instruments, Abingdon, UK) in the range of 400–1000 nm.

### 2.2. Sample Collection and Preparation

The samples were collected at baseline time (at midday) and 1 h after routine cleaning at a local indoor public gym and an indoor gymnasium at Beijing Sport University. Sterile cotton swabs immersed in sterile saline solution (1 mL) were used to swab the gymnastic equipment surfaces that frequently contact the human body, covering an area of approximately 25 cm^2^. The equipment surfaces included treadmills, recumbent bikes, free weights, mats, workout benches, locker room benches, dumbbells, and an elliptical machine. The samples were transferred into sterile centrifuge tubes and then stored at 4 °C before processing. Two mats were purchased from a local store and inoculated with *S. Aureus.* One mat was cleaned with an alcohol spray bottle every day, while another one did not undergo any cleaning; then, both of them were stored at room temperature for a week. The samples were swabbed once a day according to the collecting method, and the collected sample was directly added onto the biosensor for detection. Pathogen quantification was performed using qPCR at the same time.

### 2.3. Preparation of Bacteria Suspensions with Different Concentrations

The *E. coli*, *S. epidermidis*, and *S. aureus* standard bacteria were aseptically inoculated into Luria–Bertani (LB) broth and cultured overnight at 37 °C under continuous shaking at 200 rpm/min to achieve approximately 10^9^ CFU/mL (OD600 ≈ 1.0). Bacteria concentrations were determined via the plate count method, where 10 µL of an appropriate dilution was plated on Mueller–Hinton agar. Colony counts were expressed as colony-forming units (CFU). In total, 100 µL of the original bacteria suspensions were transferred into a sterile centrifuge tube containing 900 µL of sterile PBS solution, followed by vortex mixing to prepare a 1:10 dilution. Serial ten-fold dilutions were performed repeating this procedure with a strict adherence to aseptic technique. Finally, bacteria suspensions were diluted to target concentrations (10^0^~10^7^ CFU/mL) for the next experiments.

### 2.4. Fabrication of Nanochain-Based Biosensors

The biosensors were fabricated by following a procedure developed in a previous report [32]. Briefly, a silicon wafer (1 × 1 cm) underwent ultrasonic cleaning in deionized water three times, followed by plasma treatment (15 W, 15 s). The carboxyl-modified PS nanoparticle solution was centrifuged (3000 rpm/min for 20 min) to remove surfactants and other impurities, and was then used to prepare the nanoparticle solution. A total of 6 μL of the nanoparticle solution (2 mg/mL) containing 1 mg/mL SDS was dripped onto the silicon surface, which was then covered with a pre-designed template. The nanochains were printed onto the silicon surface with the evaporation of a solvent (water) at 50 °C for 1 h. Next, the wafer with large-area nanochains was placed in an oven (105 °C) for 45 min to enhance the structural integrity of the nanochains. To enable the efficient loading of specific antibodies, the silicon wafer with nanochains was immersed in 3 mg/mL NHS and 10 mg/mL EDC solution for 30 min to activate carboxyl groups on the surface of the PS nanoparticles. The silicon surface with nanochains was incubated in a 30 µg/mL *S. aureus* antibody solution, which was diluted with PBS at 25 °C for 2 h then rinsed with deionized water three times. The *S. aureus* antibodies were immobilized on the PS nanoparticles via covalent bonds formed between their amine groups and the carboxyl groups on the nanoparticles. Then, the biosensor surface was blocked with 1% BSA at 25 °C for 30 min to reduce nonspecific binding and then rinsed with deionized water three times. Subsequently, the biosensor was prepared for *S. aureus* detection and analysis.

### 2.5. Specific Detection of S. aureus Through Biosenors

The 50 μL bacterial sample was dropped onto the biosensor surface and incubated at 37 °C for 10 min to facilitate bacterial capture. Then, the biosensor was rinsed three times with deionized water to remove nonspecific bound bacteria. The optical changes were studied under an optical microscope (Nikon, LV100) with a 20 × objective lens (NA = 0.4) and a CCD (DS-Ri2). The test reporting could also be completed using a mobile phone with a microscope mode of 60 × magnification. Throughout the experiment, the biosensor was illuminated by an incident light which was perpendicular to the orientation of the nanochains, with an incident angle of 70°. Critically, the incident light intensity and exposure time should be kept constant throughout this experiment. Due to intense light scattering, the nanochains on the biosensor appeared bright yellowish-green, while the background remained completely black. The scattering spectra of nanochains detecting negative and positive samples were measured using confocal dark-field microscopy, which contained a monochromator and a water-cooled CCD detector in the optical range 400–1000 nm. After the optical measurements, the corresponding sample was characterized using SEM.

### 2.6. DNA Extraction and qPCR Detection of S. aureus

Genomic DNA was extracted using the QIAamp DNA Micro Kit (Cat. No56304) according to the manufacturer’s protocol. DNA concentration and purity were quantified using a NanoDrop™ One Microvolume UV-Vis Spectrophotometer (Thermo Fisher Scientific, Waltham, MA, USA). Specific primers targeting the femA gene were designed according to the reported lecture [33]: femA Forward: 5′-AACTGTTGGCCACTATGAGT-3′; Reverse: 5′-CCAGCATTACCTGTAATCTCG-3′ (Amplicon size: 306 bp). Primers were synthesized by Sangon Biotech (Shanghai) Co., Ltd. (Shanghai, China). The qPCR detection was performed using PowerUp™ SYBR™ Green Master Mix (Thermo Fisher Scientific, Waltham, MA, USA, Cat. No. A25742) with the following 20 μL reaction mix: 10 μL PowerUp™ SYBR™ Green Master Mix (Thermo Fisher Scientific, Waltham, MA, USA), 8.2 μL nuclease-free water, 0.4 μL Forward primer (10 μM), 0.4 μL Reverse primer (10 μM), and 1 μL template DNA. Thermal cycling conditions were 95 °C for 10 min (initial denaturation); 40 cycles of 95 °C for 15 s (denaturation); and 60 °C for 1 min (annealing/extension). Plasmid DNA standards were serially diluted (10-fold) in sterile nuclease-free water. Each dilution was analyzed in triplicate under optimized qPCR conditions, with nuclease-free water serving as the negative control. Standard curves were generated by plotting cycle threshold (Ct) values (*Y*-axis) against the logarithm of the starting template copy number (*X*-axis). Melt curve analysis was simultaneously performed.

### 2.7. Data Processing

The optical images of the biosensor captured through microscopy were analyzed using Image J software (National Institutes of Health, Bethesda, MD, USA) to quantify red (R), green (G), and blue (B) channel intensities of the nanochains. Nine fixed-length regions of nanochain images with the same length were selected for intensity analysis per sample. Following the recording of maximum intensity values per region, mean values with standard deviation (SD) were calculated for each color channel. To ensure data reliability, all data were obtained from three independent replicates. These quantitative metrics enabled the comparative analysis of nanochain color changes before and after *S. aureus* binding.

### 2.8. Statistical Analysis

Each measurement was performed three times and displayed as mean ± SD. The linear fitting of data was accomplished in Origin 2024 software. The significance of the differences in color intensities was assessed using a two-tailed Student’s *t*-test. The details of the significance level were * *p* < 0.05, ** *p* < 0.01, *** *p* < 0.001, and **** *p* < 0.0001.

## 3. Results and Discussion

### 3.1. Preparation of Nanochain-Based Biosensors for Rapid Detection of S. aureus

**Figure 1:**
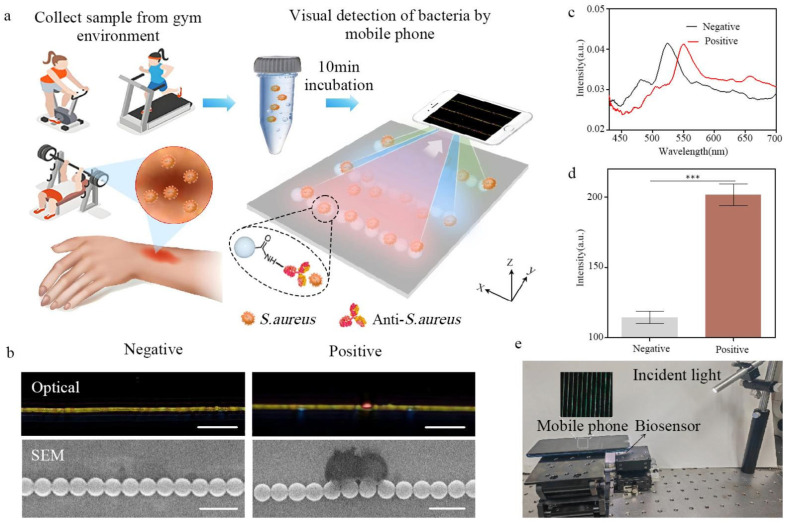
(**a**) Schematic diagram of the nanochain-based biosensors for rapid detection of *S. aureus* in gym environment. The samples collected from gym environment are dropped onto the biosensor surface and incubated at 37 °C for 10 min to facilitate bacterial capture. The target bacteria bound to the nanochains can produce distinct optical signals, which are directly observed by a mobile phone. (**b**) The optical and SEM images of nanochains that detect *S. aureus*-negative and -positive samples. (Scale bars: optical images, 10 μm; SEM images, 1 μm). (**c**) Measured scattering spectra of nanochains detecting *S. aureus*-negative (black) and -positive (red) samples. (**d**) Significant color intensity changes in the nanochains that detect *S. aureus*-negative and -positive samples in the red channel (*** *p* < 0.001). (**e**) Portable detection of *S. aureus* with a mobile phone. Insert was the optical image taken by the mobile phone. Scale bar: 100 μm.

Figure 1 illustrates the detection principle of the nanochain-based biosensor sensor for detecting *S. aureus.* Firstly, large-scale nanochains, consisting of carboxyl-modified PS nanoparticles with a diameter of 500 nm, were printed onto the hydrophobic silicon surface with the assistance of a pre-designed template [34]. Secondly, *S. aureus*-specific antibodies were immobilized on the nanochains via amide bonds formed between the amine groups of the antibodies and the carboxyl groups on the nanoparticle after being activated by NHS/EDC solutions. Next, 50 μL of the sample collected from the gym environment was added to the biosensor. Target bacteria (*S. aureus*) were captured by the specific antibodies on the nanochains, thus inducing visualized optical changes that can be recorded through optical microscopy or even a mobile phone [35] (Figure 1a). The optical images and corresponding SEM images demonstrated the effective capturing of *S. aureus* by the biosensor (Figure 1b). Specifically, the experimental setup consists of optical microscopy and a white light source, with the incident angle of the light source set at 70°. When the incident light was perpendicular to the nanochains, two resonance peaks were observed in the visible region. By taking advantage of capillary flow-assisted preconcentration with solution evaporation [32,36], this biosensor represents a high sensitivity and shortens the detection time to 10 min. With *S. aureus* binding to the nanochains, a strong near-field reconfiguration induces remarkable optical changes in both the color and intensity of scattered light, resulting in a distinct spectral shift within the visible range (Figure 1c). Notably, in addition to the use of a specific *S. aureus* antibody to capture target bacteria, the samples were rinsed three times with deionized water after incubation to remove non-specific binding contaminants and thus ensure detection specificity. The quantitative analysis of optical intensity through Image J software reveals that the red channel intensity of bacteria binding sites on the nanochains was significantly higher than that on non-binding sites (Figure 1d). To meet the demand for environmental pathogenic bacteria detection and monitoring, particularly in gym environments, we developed a portable detection system composed of a smartphone and a white light source, as shown in Figure 1e. After testing *S. aureus*-positive samples (10^4^ CFU/mL), a distinctive color change was observed in the images recorded by the smartphone (Figure S1). These results confirmed the feasibility and potency of this biosensor for on-site pathogen screening in gym settings.

### 3.2. S. aureus Detection Capability of Nanochain-Based Biosensor

**Figure 2:**
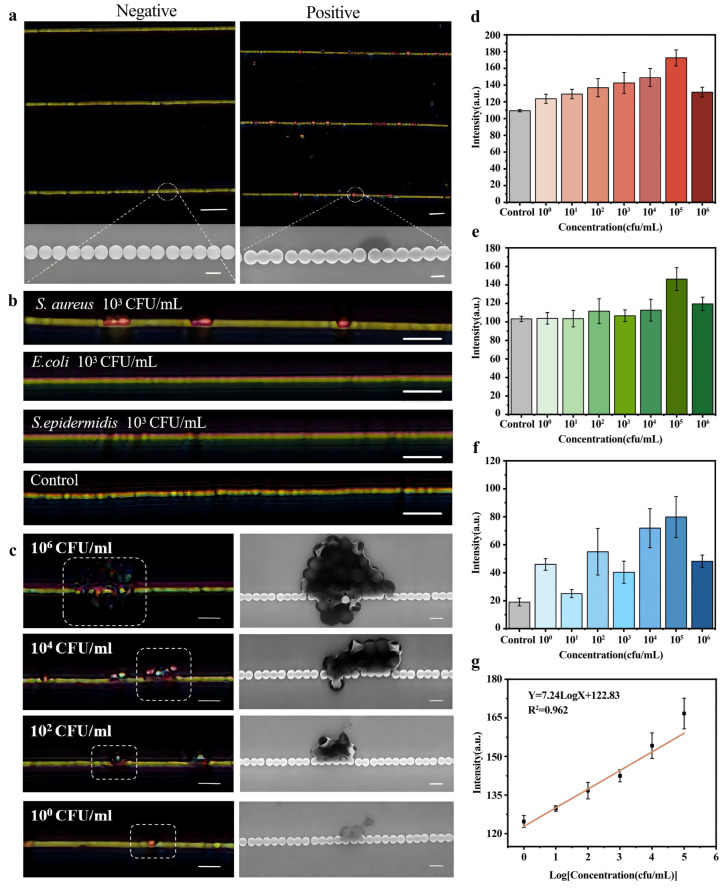
(**a**,**b**) The optical images of large-scale nanochains for detection of negative and positive samples, and corresponding SEM images (scale bars: optical images, 10 μm; SEM images, 1 μm). (**c**) The optical and SEM images of nanochains for detection of *S. aureus* with different concentrations, ranging from 10^0^ cfu/mL to 10^6^ cfu/mL (scale bars: optical images, 10 μm; SEM images, 1 μm). (**d**–**f**) the image intensity of the nanochains in the red, green, and blue channels. (**g**) Linear fitting diagrams of biosensors for detecting *S. aureus* at different concentrations.

Under the optimal conditions reported in the previous study [32], the detection capability of the biosensor for different concentrations of *S. aureus* was investigated. Figure 2a shows optical images of a negative sample (PBS solution) and a positive sample with a concentration of 10^3^ CFU/mL, which was confirmed using SEM images. Once candidate bacteria are captured, the image color of nanochains changes from yellowish-green to red and is recorded through optical microscopy with a 20 × lens, while no changes are represented when a negative sample is added onto the biosensors. Then, *E. coli* and *S. epidermidis* with concentrations of 10^3^ CFU/mL were used to validate the specificity of the biosensor (Figure 2b). No color changes happened on the nanochains when *E. coli* and *S. epidermidis* samples were added onto the biochips which were incubated with *S. aureus* antibodies; these results demonstrate the high specificity of this biosensor to detect *S. aureus.* The quantification capability of this biosensor was analyzed by adding *S. aureus*, with concentrations ranging from 10^0^ CFU/mL to 10^6^ CFU/mL (Figure S2). Optical and SEM images showed that the number of bacteria captured by the nanochains rises rapidly with increasing concentrations of *S. aureus*, thus leading to a larger range of color changes represented by the nanochains, and the limit of detection (LOD) is 1 CFU/mL (Figure 2c and Figure S2, Supporting Information). Interestingly, when the concentration of *S. aureus* reaches 10^6^ cfu/mL, aggregation and clustering phenomena were observed for the nanochains, which block the scattered light, causing the binding area to appear darker. This phenomenon may relate to the biological behavior of the bacteria. The surface of *S. aureus* contains Clumping Factor A (ClfA), which can bind fibronectin or other ligands on surrounding bacteria cells, thus leading to mutual aggregation and the formation of clusters [37]. These image colors of the biosensor were analyzed using Image J software, including the red, green, and blue channel intensities (Figure 2d–f). Specifically, nine uniformly distributed regions on nanochains of the same length (100 μm) were selected for optical intensity analysis (Figure S3i). The R-channel intensity in the bacterial binding regions was significantly higher than that in the non-binding regions, indicating that the biosensor enables the sensitive detection of *S. aureus* in gym environments (Figure S3ii). Three nanochains were measured to calculate the average intensity and standard deviation to ensure reliability and reproducibility (Figure S2). Importantly, the color intensity change of the nanochains is correlated with the number of binding bacteria. Thus, a linear relationship can be established between the average R signal intensity (y) and the concentration of bacteria samples (x), ranging from 10^0^ to 10^5^ CFU/mL, with an equation of y = 7.24LogX + 122.83, and the coefficient of determination R^2^ was 0.962 (Figure 2g). Meanwhile, *S. aureus* samples with concentrations ranging from 10^2^ cfu/mL to 10^8^ cfu/mL were analyzed using qPCR for confirmation, and the standard curve was determined for quantification (Figure S3).

### 3.3. Rapid Detection of S. aureus in Gym Environments

**Figure 3:**
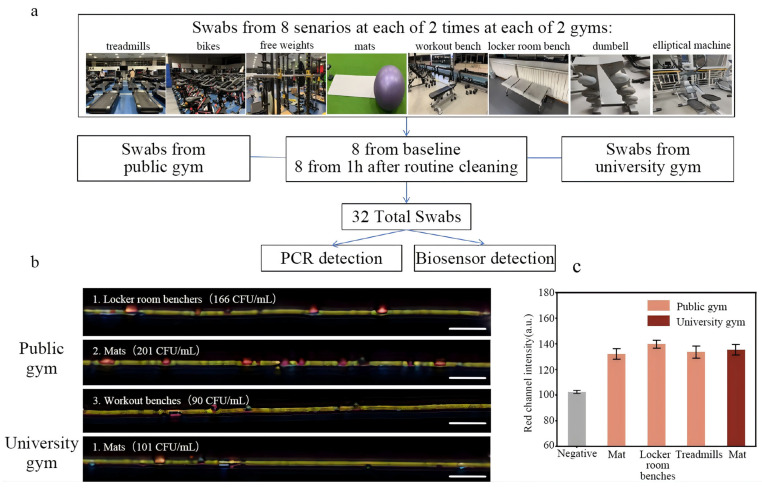
(**a**) Workflow of the sample collection from two gyms. (**b**) The optical images of nanochains after detection of positive samples collected from private gym and university gym. (**c**) Comparison of the color intensity changes in the nanochains detection of positive and negative samples in the red channel.

As shown in Figure 3a, a total of 32 swab samples were obtained from 8 scenarios in 2 separate gyms on 2 separate occasions according to the gym’s standard cleaning practices. All samples were detected using the biosensor (Figure S5) and confirmed through qPCR (Figure S6). In total, 4 of 32 samples were *S. aureus*-positive, and all positive samples were collected at a baseline time, while no positive sample was observed in the samples that were collected at 1 h after routine cleaning. Three positive samples were collected on the mats, workout benches, and locker room benches in a public gym, and one positive sample was collected on the mats at Beijing Sport University gym (Figure 3b,c). These results demonstrated the applicability of this biosensor for detecting *S. aureus* in the gym environment, and highlight the importance of routine cleaning in gym management.

### 3.4. Real-Time Monitoring of S. aureus in Gym Environments

**Figure 4:**
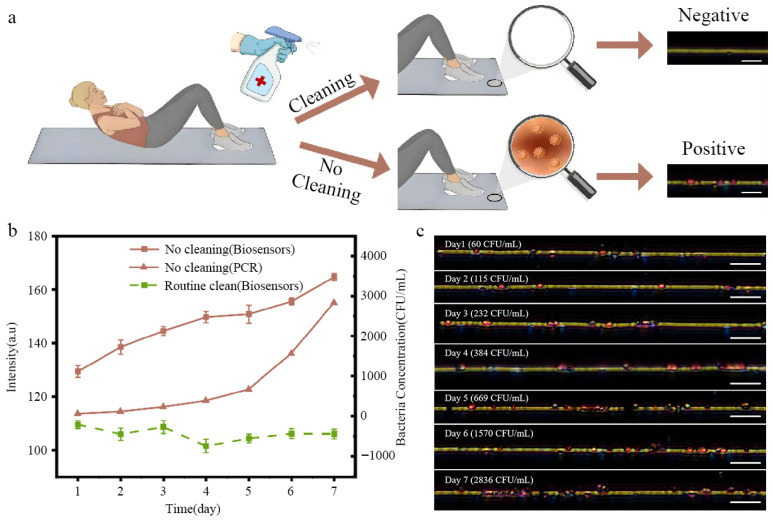
(**a**) Schematic of *S. aureus* development in gym environment. (**b**) Concentrations of *S. aureus* were monitored using biosensors and PCR for the surface of mats over one week. (**c**) Corresponding optical images of nanochains after detection of *S. aureus* during one week.

In practice, the real-time monitoring of pathogens in indoor environments with high human traffic, such as gyms, plays a crucial role in market supervision and daily management. We chose two mats used by several users and inoculated with *S. aureus*. One mat was cleaned with an alcohol spray bottle every day, while another one did not receive any cleaning; then, both of them were stored at room temperature (25~30 °C) for a week (Figure 4a). The samples were swabbed once a day, and the collected samples were directly added onto the biosensor for detection. *S. aureus* was successfully identified by the biosensor and produced visible color changes, confirmed using PCR (Figure 4b,c and Figure S7). There was no significant color change from the nanochains in the routine cleaning group, while the red signal intensity increased continuously during a week in the no cleaning group. These results underscore the significant importance of routine cleaning and real-time pathogen monitoring in indoor gym environments.

## 4. Conclusions

This study proposed a simple, rapid, culture-free, and operator-friendly pathogenic bacteria detection chip that enables the visualizable detection and monitoring of *S. aureus* in gym environments. The technology involves the robust printing of large-area nanochains onto a silicon substrate, followed by chemical conjugation with specific antibodies. Distinct color changes can be observed in optical images, allowing for a direct analysis once target bacteria are captured. This biosensor achieves the rapid and sensitive identification of *S. aureus* within 10 min, demonstrating an extremely high detection efficiency. It also enables the semi-quantitative analysis of *S. aureus* over a broad linear range (0~10^5^ CFU/mL) with an ultrasensitive detection limit (LOD: 1 CFU/mL). Compared to traditional detection methods, this technique requires no bacteria cultures, fluorescent labeling, or other complex sample preparation steps. It provides a practical detection tool suitable for daily management in gym facilities and market supervision applications. Meanwhile, this biosensor holds promise for q broad application in areas including food safety, clinical medicine, pharmaceuticals, and cosmetics.

## Data Availability

The data that support the findings of this study are available from the corresponding authors upon reasonable request.

## Supplementary Materials

The following supporting information can be downloaded at https://www.mdpi.com/article/10.3390/bios15120791/s1. Figure S1: Portable detection of *S. aureus* with a mobile phone. Figure S2: Optical images of nanochains after the detection of *S. aureus* with different concentration ranging from 10^0^ CFU/mL to 10^6^ CFU/mL. Figure S3: Analysis of color changes of the nanochains after binding bacteria. Figure S4: The qPCR analysis results and corresponding standard curve of *S. aureus* samples. Figure S5: The result of biosensors detection of *S. aureus* in the samples collected from gym environments. Figure S6: The results of qPCR detection of *S. aureus* in the samples collected from gym environment. Figure S7: Optical images of real-time monitoring of *S. aureus* growth on mats with and without cleaning over 7 days. The qPCR analysis results of corresponding samples.
