# Supplementary material for: Rapid Detection of Staphylococcus aureus from Gym Environments for Health Risk Monitoring Using Printed Nanochains-Based Biosensors
Source: Biosensors (Basel). 2025 Dec 1;15(12):791. doi: 10.3390/bios15120791 (PMC12730415; doi:10.3390/bios15120791)
Supplement: Supplementary file 1 [file biosensors-15-00791-s001.zip › biosensors-3916069-supplementary.pdf]

Support information

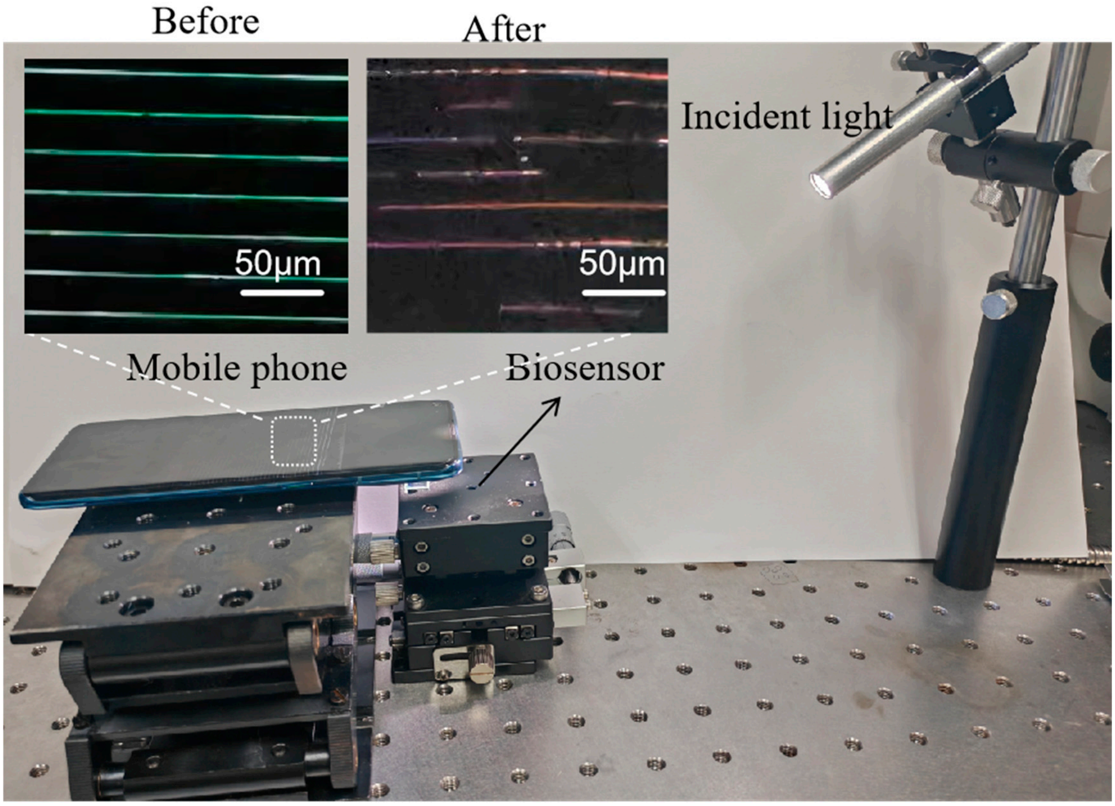

Figure S1: Portable detection of *S. aureus* with a mobile phone.

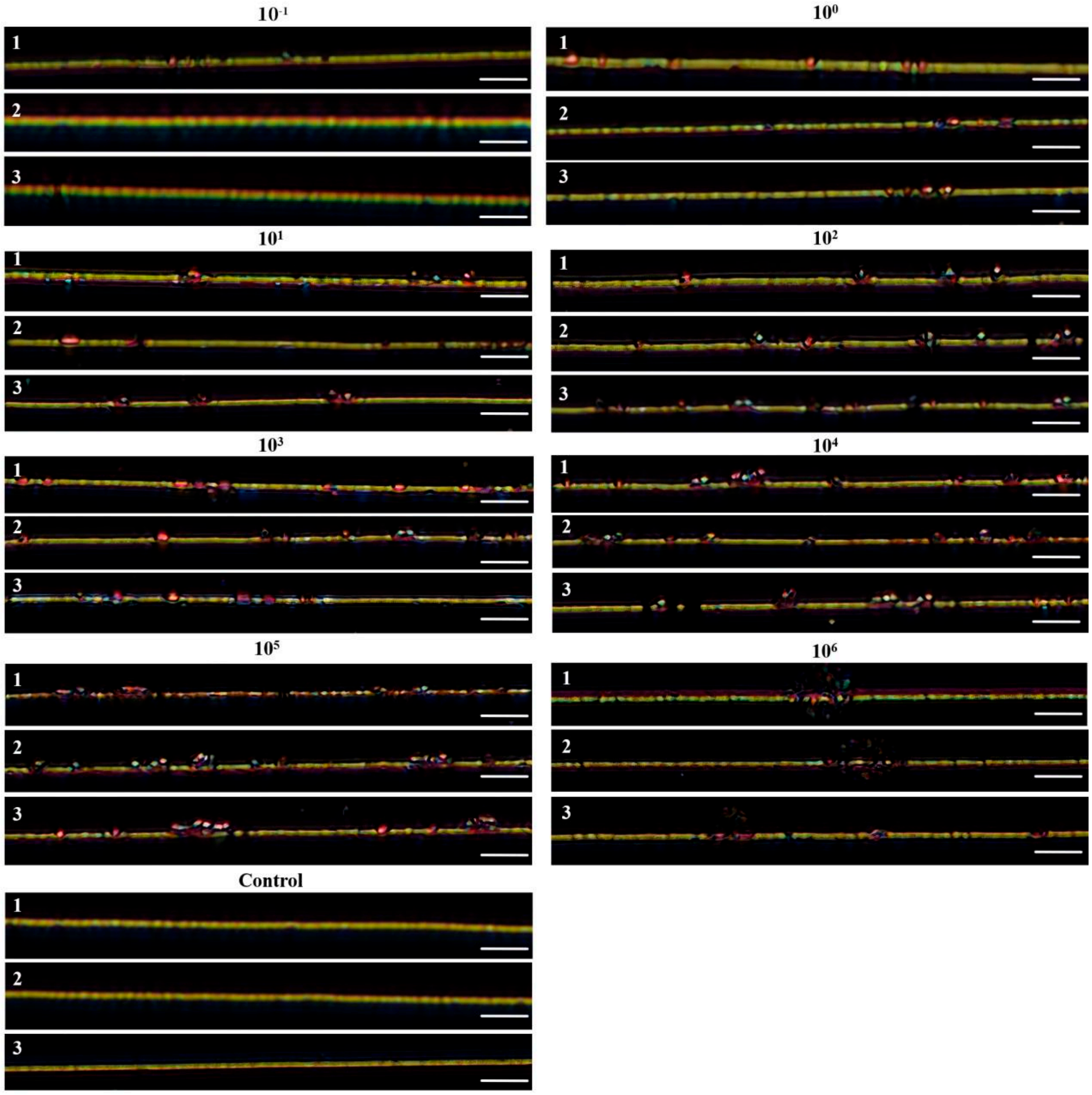

Figure S2: Optical images of nanochains after the detection of *S. aureus* with different concentration ranging from 10^0 CFU / mL to 10^6 CFU / mL. Scale bars: 10 μm.

i Optical images and regions selection

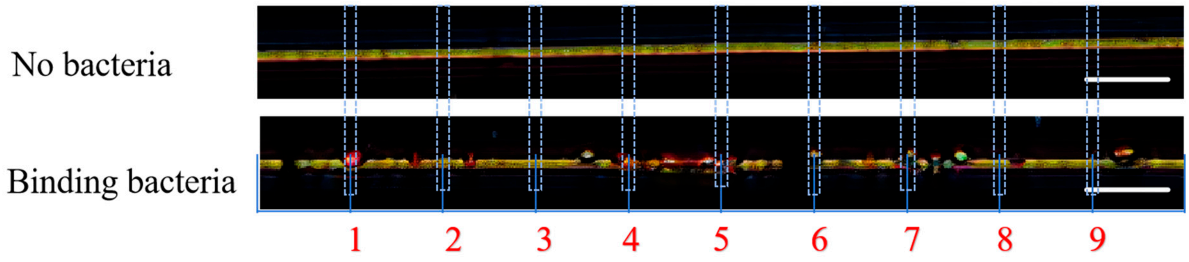

ii Intensity analysis

Region number		1	2	3	4	5	6	7	8	9	Mean	SD
No bacteria	R	105.826	110.969	108.559	100.452	100.577	102.944	113.679	108.2	113.643	107. 205	5. 124
	G	100.696	106.469	93.971	102.194	99.923	88.583	114.107	109.85	113.214	103. 223	8. 598
	B	13	14.875	24.235	21.149	12	14.361	12.179	37.6	21.607	19	8. 328
Binding bacteria	R	224.85	113.406	118.875	170.191	217	134.958	110.111	125.646	107.5	146.948	46. 017
	G	99.95	98.969	102.239	86.255	135.708	109.208	60.111	111.062	102.944	100.716	20. 226
	B	91.713	26.938	28.148	13.957	101.292	61.104	25.667	26.083	49.25	47.128	31. 410

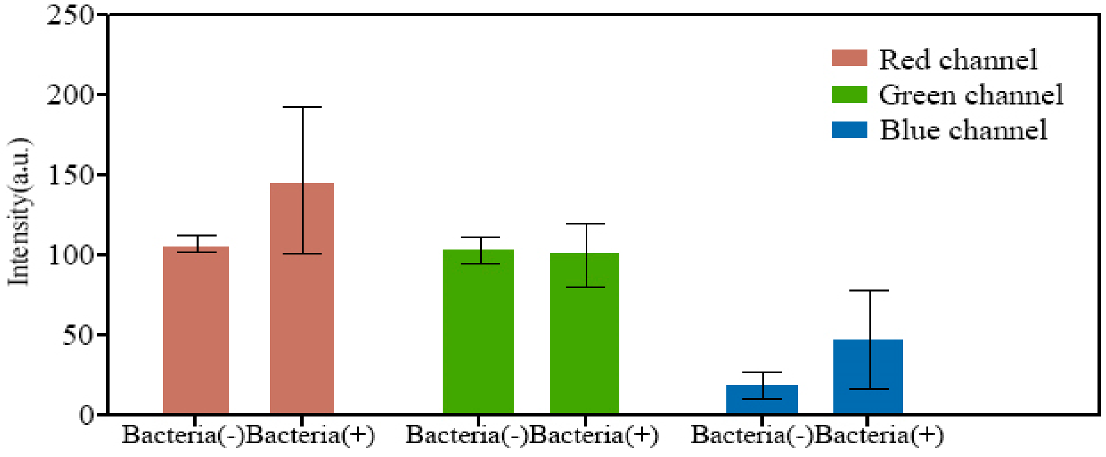

Figure S3: Analysis of color changes of the nanochains after binding bacteria. i) The optical images of nanochains with same length(100 μm) were obtained by a common optical microscopy with a 20×objective lens. nine regions (blue dotted box) at the same interval are selected on each image for analysis ii) The images were split into red (R), green (G), and blue (B) channels through Image J. The intensity of each region was recorded to calculate the average and standard deviation of the signal. The histogram was used to compare the change of each signal after binding bacteria. . In order to improve the reliability, All data in the subsequent experiments were obtained from three independent replicates.The error bar is the standard deviation(SD). Scale bars: 10 μm

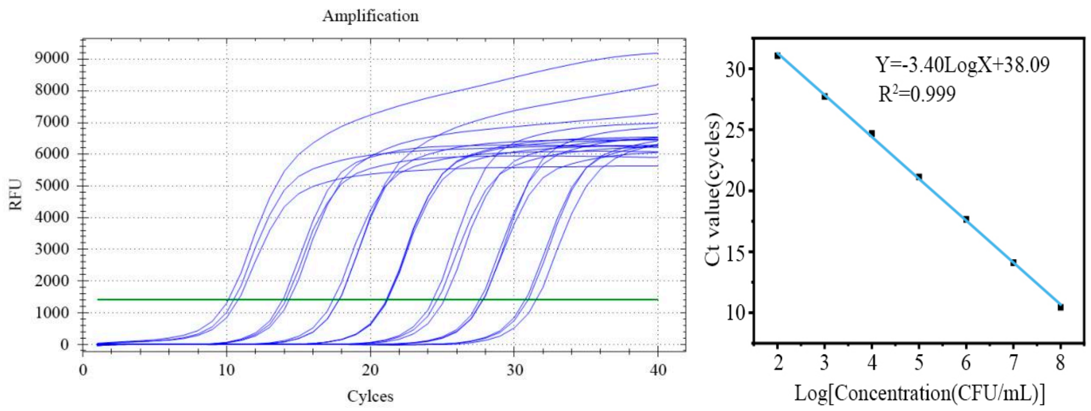

Figure S4: *S. aureus* samples (concentration ranging from 10^2 CFU / mL to 10^8 CFU / mL) were analyzed by qPCR for confirmation.The standard curve was conducted for *S. aureus* quantification.

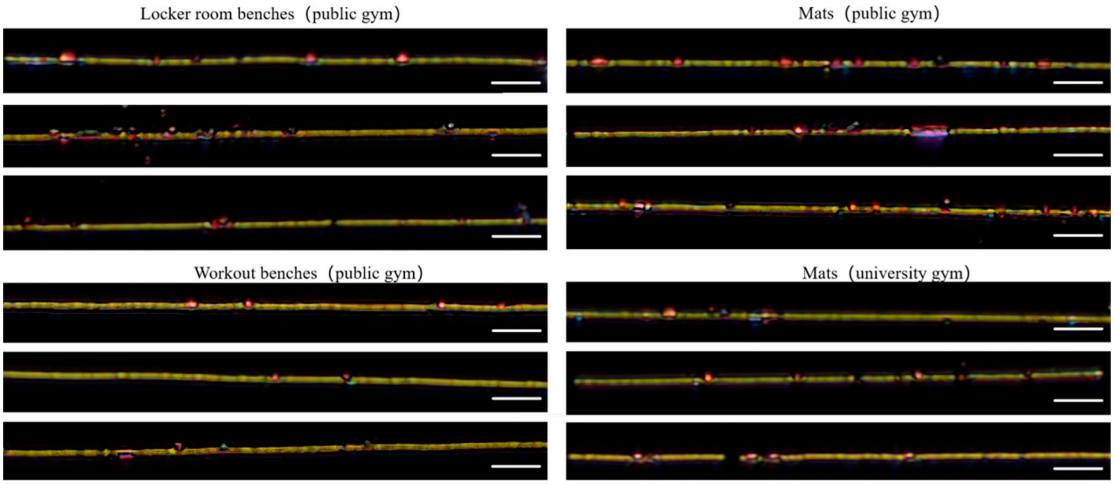

Figure S5: The result of biosensors detection of *S. aureus* in the samples collected from gym environments. Scale bars: 10 µm

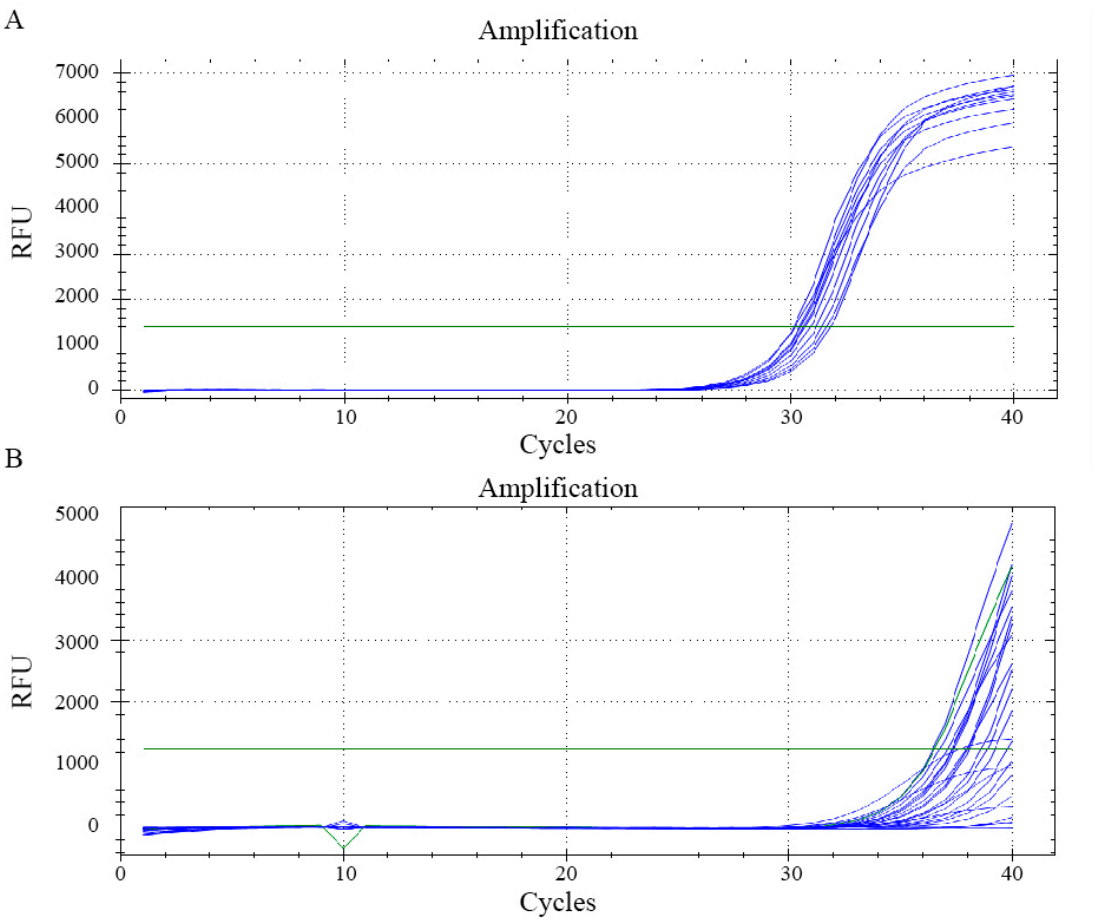

Figure S6: The results of qPCR detection of *S. aureus* in the samples collected from gym environment (A. positive sample; B. negative sample).

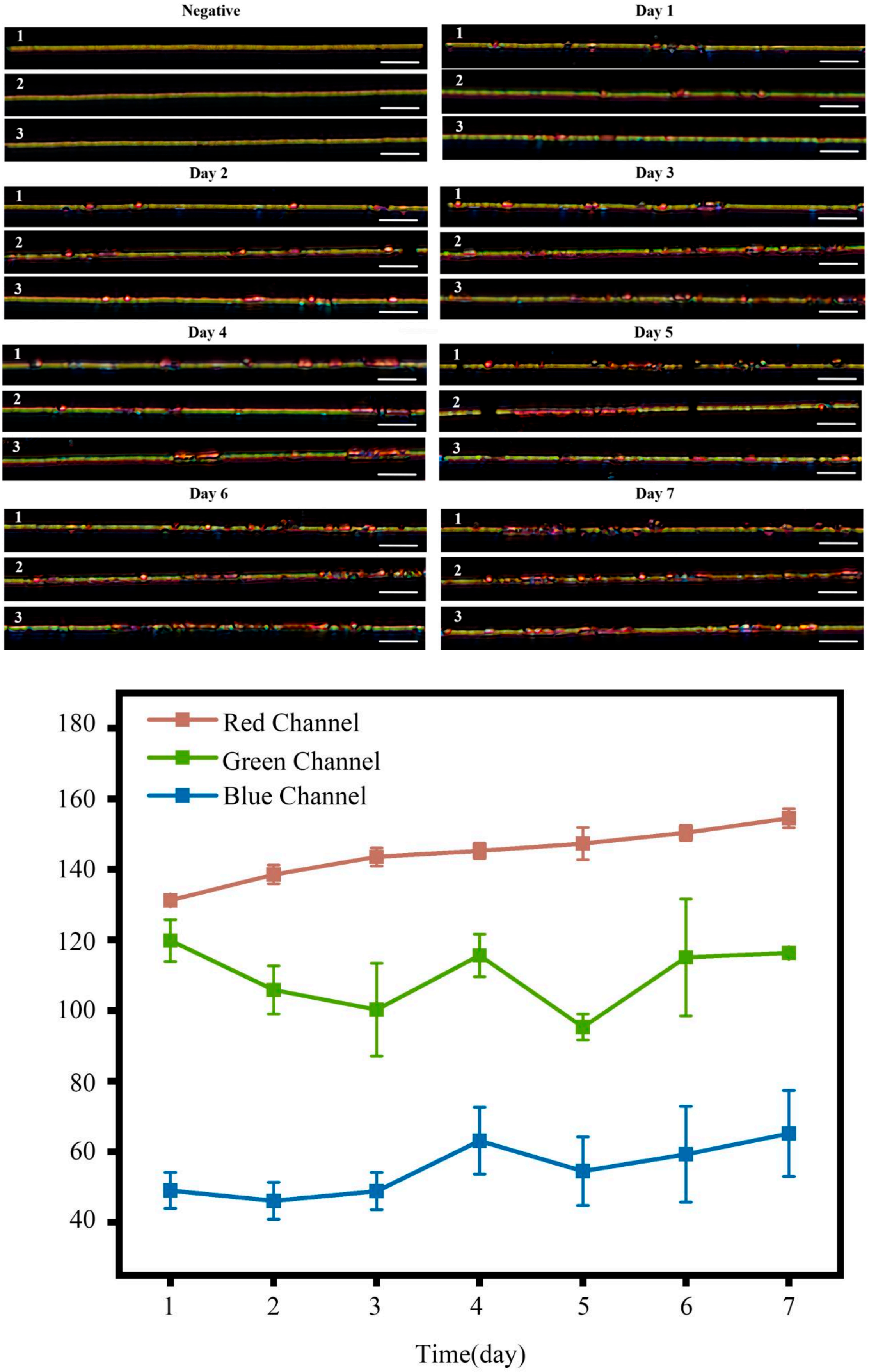
Negative
Day 1
Day 2
Day 3
Day 4
Day 5
Day 6
Day 7
1
2
3
Red Channel
Green Channel
Blue Channel
180
160
140
120
100
80
60
40
1
2
3
4
5
6
7
Time(day)

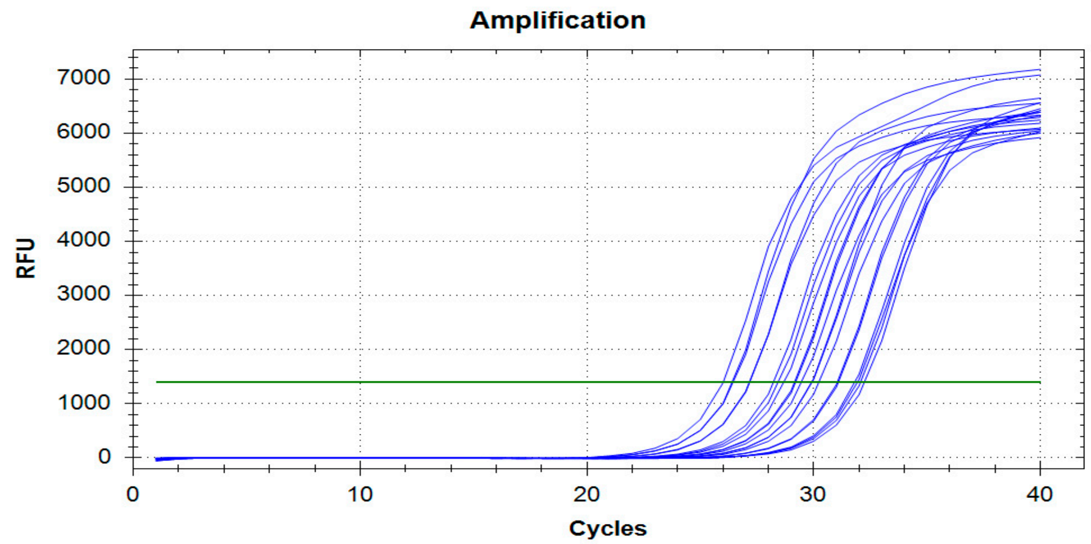

Figure S7: Optical images of real-time monitoring of *S.aureus* growth on mats with and without cleaning over 7 days. Corresponding samples were analyzed by qPCR for quantification. Scale bars: 10 μm.
